# Virtual Culturally Grounded Interventions for Substance Use in Urban American Indian and Alaska Native Emerging Adults: A Randomized Clinical Trial

**DOI:** 10.1001/jamanetworkopen.2026.27878

**Published:** 2026-08-11

**Authors:** Elizabeth J. D’Amico, Daniel L. Dickerson, Anthony Rodriguez, David J. Klein, Virginia Arvizu-Sanchez, Kurt Schweigman, Carrie Johnson, Sam Mann, David P. Kennedy, Pierrce Holmes, Alina I. Palimaru, Ryan Brown, Nipher Malika

**Affiliations:** 1 RAND Corporation, Santa Monica, California; 2 UCLA Integrated Substance Use and Addiction Programs, Semel Institute for Neuroscience and Human Behavior, David Geffen School of Medicine, University of California, Los Angeles; 3 RAND Corporation, Boston, Massachusetts; 4 Sacred Path Indigenous Wellness Center, Long Beach, California; 5 Santa Rosa, California; 6 RAND Corporation, Arlington, Virginia

## Abstract

**Importance:**

American Indian and Alaska Native individuals report higher rates of substance use disorders and psychological distress compared with individuals from other racial and ethnic groups. However, few evidence-based, culturally grounded programs address substance use and mental health for urban American Indian and Alaska Native emerging adults.

**Objective:**

To test 2 culturally grounded virtual interventions for substance use among urban American Indian and Alaska Native emerging adults.

**Design, Setting, and Participants:**

In this randomized clinical trial, participants were recruited across the US from December 1, 2020, to October 27, 2023. Participants completed an online screening questionnaire. Eligibility criteria included (1) age 18 to 25 years; (2) living in an urban area in the US (not a rancheria or a reservation); (3) self-identification as American Indian or Alaska Native; (4) no opioid use disorder; and (5) English speaking. The participants completed baseline surveys, were randomized to 1 of 2 culturally grounded virtual interventions, and completed 3-, 6-, and 12-month surveys. Follow-up was completed January 28, 2025. Analysis was based on intention to treat.

**Interventions:**

Participants received either 3 Traditions and Connections for Urban Native Americans (TACUNA) workshops and a wellness circle, or an opioid education health and wellness cultural (HWC) workshop (usual care).

**Main Outcomes and Measures:**

Surveys focused on opioid, alcohol, and cannabis use (primary outcome) and consequences of alcohol and other drug use, mental health, cultural connection, and peer influence (secondary outcomes).

**Results:**

Among the 541 randomized participants (451 [83.2%] female; mean [SD] age, 22.1 [2.2] years), the TACUNA and HWC groups both reported decreased frequency of cannabis use (B [SE],  −0.44 [0.20] and −0.54 [0.21], respectively) and decreased rates of positive screens for alcohol (B [SE], −0.03 [0.01] and −0.02 [0.01], respectively) and cannabis (B [SE], −0.02 [0.01] and −0.03 [0.01], respectively) use disorders. Rates of time spent with peers who use alcohol and prescription opioids, clinical depression and anxiety, alcohol and cannabis consequences, and peer norms around alcohol, cannabis, prescription opioids, and heroin use improved in both groups. Only TACUNA participants reported decreases in frequency (B [SE], −0.51 [0.20]) and quantity (B [SE], −0.25 [0.09]) of alcohol use and quantity of cannabis use (B [SE], −0.07 [0.03]). In addition, TACUNA participants reported greater decreases in time spent around peers using cannabis and heroin and greater decreases of anxiety compared with HWC participants.

**Conclusions and Relevance:**

In this randomized clinical trial of 2 virtual culturally grounded interventions for substance use, virtual recruitment and intervention successfully reached urban American Indian or Alaska Native emerging adults with decreased access to resources. Both groups reduced consequences and cannabis use; however, only TACUNA participants reported decreases in quantity of alcohol and cannabis use and time spent around peers using cannabis and heroin and greater decreases in anxiety. These findings emphasize the role of bringing American Indian and Alaska Native emerging adults together to discuss ways to reduce alcohol and other drug use and socially connect with their tribal communities in the urban environment in a virtual setting.

**Trial Registration:**

ClinicalTrials.gov Identifier: NCT04617938

## Key Points

**Question:**

How do 2 culturally grounded virtual interventions for urban American Indian and Alaska Native emerging adults affect subsequent substance use 1 year later?

**Findings:**

In this randomized clinical trial of 541 participants, both the intervention and usual care groups reported decreased frequency of cannabis use. Only participants in the intervention group reported decreases in frequency and quantity of alcohol use and quantity of cannabis use.

**Meaning:**

These findings emphasize the importance of bringing urban American Indian and Alaska Native emerging adults together to discuss ways to reduce alcohol and other drug use and socially connect with their tribal communities in a virtual setting.

## Teaser

This randomized clinical trial assessed 2 culturally grounded interventions for substance use disorder among urban American Indian and Alaska Native emerging adults.

## Introduction

American Indian and Alaska Native people emphasize the importance of living a healthy balanced life that is grounded in their cultural traditions and resiliency,^1,2,3^ and work has shown that protective factors such as cultural connectedness and participating in traditional practices are associated with decreased alcohol and other drug (AOD) use and improved mental health.^4,5,6^ However, American Indian and Alaska Native people still experience health disparities. Rates of opioid overdose deaths have increased from 0.36 per 100 000 between 1999 and 2019 to 6.50 between 2019 and 2021.^7^ American Indian and Alaska Native people report higher rates of cannabis use than all other racial and ethnic groups, and 70% of American Indian and Alaska Native individuals who report past month alcohol use engage in heavy drinking.^8,9^ In addition, American Indian and Alaska Native people report 3 times the rate of psychological distress compared with Black, Hispanic, and White individuals.^10^

Historical trauma, including forced relocation from tribal homelands, has contributed to disparities, partly through cultural disconnection.^11,12^ More than 87% of American Indian and Alaska Native individuals reside outside reservations and tribal lands,^13^ yet urban American Indian and Alaska Native people are historically underrepresented in research.^12^ The urban environment poses challenges for American Indian and Alaska Native people due to geographic and social fragmentation.^14^ Although some urban American Indian and Alaska Native communities are still closely connected, many urban individuals feel culturally disconnected, as they are often unable to participate in tribal traditions given geographic distribution of their people.^14^ Previous work with urban American Indian and Alaska Native emerging adults (ie, individuals aged 18-25 years) has emphasized that supportive social networks for members who engage in traditional practices can be protective for AOD use and mental health problems.^15,16^ Further, participating in traditional practices is protective for AOD use among urban American Indian and Alaska Native young people and adults.^4,15,17,18^ However, barriers including transportation and expense make it difficult for American Indian and Alaska Native people in urban areas to access cultural experiences.^19,20^

To date, culturally grounded programs that address AOD use for American Indian and Alaska Native people living in urban areas are limited,^12^ particularly among urban emerging adults.^21^ Furthermore, no programs are available to address opioid, cannabis, and alcohol misuse among urban American Indian and Alaska Native emerging adults that integrate culturally grounded strategies with evidence-based treatment.^22^ Of note, emerging adulthood is a particularly high-risk time for increases in both cannabis and alcohol use and mental health symptoms,^23,24^ yet this age group is the least likely to obtain services.^24^ One study with American Indian and Alaska Native young people^18^ showed that 87% of participants reported lifetime use of alcohol, 76% reported lifetime use of cannabis, and 66% met criteria for polysubstance use. Thus, intervening with American Indian and Alaska Native young people during emerging adulthood is critically important.

Motivational interviewing (MI) is a collaborative, nonjudgmental approach^25^ that has been shown in numerous trials to be an effective way to address health risk behaviors among young people.^26,27^ Previous work has shown that MI interventions decrease risk behavior among racially and ethnically diverse individuals,^26,28^ including American Indian and Alaska Native people.^4,29^ For example, a prior intervention program that included most of our investigators (Motivational Interviewing and Culture for Urban Native American Youth [MICUNAY]) integrated traditional practices with MI to address substance use among urban American Indian and Alaska Native adolescents.^4^ MI has shown high acceptability and proven utility among American Indian and Alaska Native people.^22,30,31^ Furthermore, traditional practices are critical for prevention of AOD use among American Indian and Alaska Native young people, as they can confer protection given their focus on community connectedness.^1,32,33,34^

This randomized clinical trial (RCT) tested 2 culturally grounded interventions for urban American Indian and Alaska Native emerging adults. The first intervention integrated MI, an evidence-based treatment to address substance use,^25^ and traditional practices to address opioid, alcohol, and cannabis use and thereby increase cultural connection (Traditions and Connections for Urban Native Americans [TACUNA]). The second intervention focused on opioid education and health and wellness cultural workshops (HWC).^35^ This study was developed in collaboration with our Elder Advisory Board (EAB) (including V.A.S., K.S., and C.J.) and our community partner, Sacred Path Indigenous Wellness Center (SPIWC).

Our study was designed prior to COVID-19 as an in-person study to be conducted in California. However, before the RCT began, stay-at-home orders occurred, and organizations turned to virtual and telehealth programming. We redesigned in-person workshops to be virtual^20^ and opened the RCT to urban American Indian and Alaska Native emerging adults across the US. We tested the 2 culturally grounded virtual interventions and examined effects on AOD use and consequences, anxiety and depression, cultural connection, perception of peer use, and time spent around peers who use substances during 12 months of follow-up.

Given the focus in TACUNA on ways to decrease AOD use, leveraging healthy social networks and traditional practices, we hypothesized that TACUNA participants would report greater decreases in substance use and consequences, perception of peer use, and time spent around peers who use substances and greater improvements in levels of anxiety and depression and cultural connection than the health and wellness condition. We recognize that cultural connection may also mediate substance use and mental health outcomes, for example, with the intervention increasing cultural connection, which could then be associated with decreases in substance use and improvements in mental health. For the current study, however, we specifically focused on direct effects of the intervention.

## Methods

This randomized clinical trial was approved by the Institutional Review Board of RAND Corporation and the Urban Intertribal Native American Review Board; all participants provided verbal informed consent. The trial protocol has been published previously^35^ and is also provided in Supplement 1. The study followed the Consolidated Standards of Reporting Trials (CONSORT) reporting guideline.^36^

### Participants

Participants were recruited via social media using QR codes on our website and ads on Instagram (Meta Platforms) and Facebook (Meta Platforms) from December 1, 2020, to October 7, 2023; interested participants completed an online screening questionnaire.^37^ Eligible participants were contacted, consented, completed a baseline survey, and were randomized to receive either 1 virtual workshop or 3 virtual workshops and a Wellness Circle (WC) program using a permuted block randomization schedule, with a block size of 6.^35^ Eligibility criteria included (1) age 18 to 25 years; (2) living in an urban area in the US (not on a rancheria or a reservation); (3) self-identification as American Indian or Alaska Native; (4) no opioid use disorder per the Rapid Opioid Dependence Screener^38^; and (5) English speaking. Of the 541 participants who completed the baseline survey, 503 (93.0%) completed the 3-month survey, 508 (93.9%) completed the 6-month survey, and 496 (91.7%) completed the 12-month survey, with all participants completing the final follow-up by January 28, 2025. Participants were paid $40 for completing the baseline, $60 for the 3-month, $80 for the 6-month, and $100 for the 12-month surveys.

### Interventions

American Indian and Alaska Native communities are often concerned that randomization does not provide benefits to the entire community; thus RCTs are often perceived as unacceptable and unfair.^12^ Based on previous work^4,31^ and collaborations with SPIWC, our EAB, and state and local American Indian and Alaska Native organizations, we ensured that all participants received some form of culturally appropriate programming. We collaborated with SPIWC and our EAB to develop an opioid, alcohol, and cannabis prevention intervention program for urban American Indian and Alaska Native emerging adults focusing on feasibility and sustainability.^35^ We sought to address challenges faced by urban American Indian and Alaska Native emerging adults due to AOD use and disconnection from culture and supportive networks. Pilot work provided formative information on integrating MI to address AOD use with culture and helped us virtually test workshops, which were well received.^20^

The TACUNA intervention consisted of three 2-hour virtual group workshops. The first hour used MI to address AOD use and social network choices, including ways to find support in one’s network and decrease risk; the second hour integrated American Indian and Alaska Native culture, including discussion of storytelling for ones’ American Indian and Alaska Native identity, Native American cooking, and a prayer and sage ceremony.^35^ A key part of TACUNA was to both talk about and engage in different traditional practices in the group setting. For example, as part of workshop 2, participants learned about traditional Native foods, discussed ways to incorporate traditional foods into their lives, and were provided a recipe and cooking ingredients to make Three Sisters Stew together during the virtual workshop. TACUNA participants also received a 1-hour virtual WC program, which occurred monthly. WCs were advertised via social media, so anyone could attend, although TACUNA participants were specifically told about WCs. WC activities focused broadly on wellness and culture, such as drumming and traditional dances, and discussion of the Medicine Wheel and how to incorporate these teachings into one’s life.^35^

The control condition comprised a 2- hour HWC workshop focused on an overview of opioids and pain self-management based on prevention materials recommended by the National American Indian and Alaska Native Addiction Technology and Transfer Center of the Substance Abuse and Mental Health Services Administration.^39^ Workshops were held weekly, and participants had 3 months after their baseline survey to complete either all TACUNA workshops and a WC or the HWC workshop. Participants in both groups received handouts and materials (eg, cooking ingredients and sage for TACUNA) via mail. TACUNA and HWC workshops were led by American Indian and Alaska Native facilitators trained on each protocol. TACUNA facilitators were also trained on MI. In January 2024, workshops in both conditions were shortened to 1 hour based on feedback from participants to decrease Zoom time as in-person activities started to open after the pandemic, making attending more feasible.^21^ We recorded all workshops for TACUNA and HWC groups. Weekly supervision was provided by 2 investigators (E.J.D. and D.L.D.).

### Adverse Events

The most frequent adverse event during this project was related to responses on the final question on the 9-item Patient Health Questionnaire.^40^ This question asked whether in the past 2 weeks the participant had “thoughts that you would be better off dead, or of hurting yourself.” If a participant reported a response greater than “not at all” to this question, the participant was called by a clinical psychologist or psychiatrist to check to ensure they were safe and whether they needed additional support per the Institutional Review Board–approved data safety and monitoring plan. This could occur at any of the 4 survey time points. During the course of this study, 76 participants in the TACUNA group and 75 participants in the Health and Wellness group endorsed a response greater than not at all on this question across all 4 survey time points.

### Measures

#### Demographic Characteristics

We collected self-reported age, sex at birth, gender, sexual orientation, mother’s educational level, and genders of past sexual partners. Based on answers, we defined sexual and gender diverse as any sexual orientation other than straight or heterosexual, sex at birth as something else, gender identity as gender fluid or something else, transgender identity, history of same-gender sexual intercourse, or discordance between sex at birth and gender identity.

#### Opioid, Alcohol, and Cannabis Use

For prescription opioid misuse, we used language created as part of the HEAL (Helping to End Addiction Long-Term) prevention cooperative focused on “using [opioids] in any way a doctor or medical provider did not tell you to use them.” For prescription opioid misuse and heroin, alcohol, and cannabis use, items assessed the number of times used in the past 3 months; responses (none, 1, 2, 3-5, 6-9, 10-19, 20-30, or ≥31 times) were recoded to estimate the actual number of times used (0, 1, 2, 4, 7, 14, 25, and 31, respectively). For those who used alcohol, we assessed maximum number of alcoholic drinks consumed on any day in the past 30 days. One alcoholic drink (beverage) was defined as 1 regular size can or bottle of beer or wine cooler, 1 glass of wine (5 ounces), 1 mixed drink, or 1 shot glass (1.5 ounces) of liquor. For participants who used cannabis, we assessed quantity of cannabis (flower or bud) they normally used from 0.25 to more than 5.00 g.^41^ Alcohol use disorder was measured with the 3-item Alcohol Use Disorder Identification Test, and screening was positive if the response sum was at least 3 for females or at least 4 for males. Cannabis use disorder was measured with the Cannabis Use Disorder Identification Test–Short Form, and screening was considered positive if the response sum was at least 2.

#### Opioid, Alcohol, and Cannabis Use Consequences

Participants reported consequences in the past 3 months (eg, missed school, work, or other obligations) for each substance used (8, 10, and 13 items respectively).^42^ Survey responses (never, 1, 2, 3-5, 6-9, 10-19, or ≥20 times) were recoded to estimate actual number of times (0, 1, 2, 4, 7, 14, and 20) and summed. Consequences were defined as missing for participants who did not report any use of that substance.

#### Mental Health

Depression and anxiety in the last 2 weeks were assessed using a sum from the 9-item Patient Health Questionnaire^40^ and the 7-item Generalized Anxiety Disorder Scale^43^ (scores range from 0 [not at all] to 3 [nearly every day]). Outcomes focused on whether someone did (or did not) report clinically meaningful depression or anxiety (ie, scores ≥10).

#### Cultural Connection

We measured cultural connection using the 24-item Sense of Community Index–2, which spans reinforcement of needs, membership, influence, and shared emotional connection^44^ (scores range from 0 [not at all] to 3 [completely]). Mean scores were computed.

#### Peer Influence

Participants reported the percentage of people their age they thought used alcohol, cannabis, prescription opioids, and/or heroin monthly (range, 0-100%, scored from 0 to 10 [100%]). They also reported how much time they spent around people who used alcohol, cannabis, prescription opioids, or heroin. Scores included 0 (never), 1 (hardly), 2 (sometimes), and 3 (often).

#### Fidelity

The principal investigators (E.J.D. and D.L.D.) on the study listened to the digital recording for every workshop and provided weekly supervision to the facilitators. We measured fidelity by coding 20% of workshops with a checklist from previous group work that listed the different components of each workshop (eg, discussion of path of choices) (eAppendix in Supplement 2).^4^ For TACUNA, we also coded MI fidelity using the Motivational Interviewing Treatment Integrity global scales, version 3.1.1,^45^ validated for use in group settings.^4^ Scores of 4 or higher on global scales (evocation, collaboration, autonomy-support, empathy, and direction) indicated MI competency.

### Statistical Analysis

We evaluated comparability of treatment arms on baseline measures to ensure equivalence of groups. Fisher exact tests compared groups for categorical characteristics and independent samples *t* tests compared groups for continuous characteristics. Primary outcomes consisted of substance use. Secondary outcomes included consequences, mental health, cultural connection, and peer influence. Analyses were based on intention to treat. At baseline, outcomes were never missing except for 2 outcomes where the outcome was missing for 1 person. Data for age, sex, and sexual and gender diversity were never missing. We used SAS, version 9.4 (SAS Institute Inc) for the aforementioned analyses.

We estimated a multigroup latent growth model (LGM) in a structural equation modeling framework with Mplus, version 8.9 for measures examined longitudinally across all 4 time points using robust maximum likelihood estimation, which can accommodate missing data, handle nonnormality, and provide unbiased and consistent estimates.^46^ In LGM, the model intercept reflects the estimated value of the outcome when the covariate is equal to zero and thus represents a baseline level. The slope reflects rate of change over time. LGMs were evaluated using conventional model fit criteria: χ^2^, root mean square error of approximation no greater than 0.08, and comparative fit index no greater than 0.95. Growth parameters (intercepts and slopes) were statistically tested using *z* tests. The multigroup LGM framework estimates LGMs in each group separately and simultaneously to characterize longitudinal change in each group as well as provide the ability to statistically test observed differences in trajectories by imposing and testing parameter equality constraints (eg, cannabis use slope to be equal between TACUNA and HWC conditions) using the Wald test. Last, the effect of workshop length (2 vs 1 hour) was estimated using similar models but restricted to TACUNA participants who attended at least 1 workshop. As a note, several approaches and parameterizations are available for estimating intervention effects both within and outside the latent variable modeling framework. Although specifying a single LGM with intervention condition included as a time-invariant covariate represents a reasonable and commonly used parameterization, this approach implicitly imposes assumptions regarding the equivalence of residual variances, latent factor variances and covariances, measurement error structures, and functional form across intervention conditions. In contrast, the multigroup LGM framework relaxes these constraints, allowing model components to vary across intervention groups, thereby providing greater flexibility to evaluate potential heterogeneity in longitudinal processes. Accordingly, the multigroup framework was selected for the present analyses.

## Results

### Descriptive Information

The sample of 541 American Indian and Alaska Native emerging adults included 450 females (83.2%) and 91 males (16.8%), with a mean (SD) age of 22.1 (2.2) years (Table 1). Participants came from more than 200 tribes and 37 states, with highest participation rates in California, Arizona, Washington, Oklahoma, and Minnesota. Few participants reported opioid use (prescription and heroin) in the past 3 months (at baseline, 10 reported opioid use and 1 reported heroin use in the TACUNA group; 4 reported opioid use and 0 reported heroin use in the HWC group); thus, opioid use and consequences were excluded from analyses. Rates of use were similar to national norms for alcohol use (Monitoring the Future found approximately 66% in this age group report use; our study found 66%), and higher for cannabis use (eg, Monitoring the Future found approximately 30% in this age group report use; our study found 50%).^47^

**Table 1.** Baseline Participant Characteristics

Characteristic	Study group	
	TACUNA (n = 279)	HWC (n = 262)
Demographic		
Age, mean (SD), y	22.1 (2.2)	22.1 (2.2)
Sex, No. (%)		
Female	233 (83.5)	217 (82.8)
Male	46 (16.5)	45 (17.2)
Sexual and gender diverse, No. (%)	142 (50.9)	144 (55.0)
Behaviors, mean (SD)		
Alcohol use in past 3 mo	8.78 (10.43)	8.91 (10.40)
Cannabis use in past 3 mo	9.90 (12.79)	10.62 (13.04)
Alcohol negative consequences in past 3 mo^a^	7.58 (15.60)	8.89 (21.21)
Cannabis negative consequences in past 3 mo^a^	20.99 (36.69)	18.35 (31.17)
How often with people who use AOD^b^		
Alcohol	2.15 (0.88)	2.09 (0.85)
Cannabis	2.27 (0.92)	2.20 (0.97)
Opioids	0.52 (0.81)	0.43 (0.73)
Heroin	0.17 (0.47)	0.13 (0.44)
Monthly, how many peers your age use AOD?^c^		
Alcohol	7.11 (2.23)	7.32 (2.16)
Cannabis	6.53 (2.24)	6.68 (2.28)
Opioids	2.76 (2.01)	2.82 (2.10)
Heroin	1.53 (1.53)	1.59 (1.65)
Most alcoholic beverages consumed in 1 d during past 1 mo	7.13 (3.61)	7.30 (3.89)
Amount of cannabis used per day when using, g	3.04 (2.83)	2.77 (2.82)
Clinically meaningful depression, No. (%)^d^	119 (42.7)	115 (43.9)
Clinically meaningful anxiety, No. (%)^e^	107 (38.4)	100 (38.2)
AUDIT-C positive screen, No. (%)	127 (45.5)	107 (40.8)
CUDIT-SF positive screen, No. (%)	82 (29.4)	81 (30.9)
Cultural connection, mean (SD)^f^	1.62 (0.64)	1.67 (0.59)

Abbreviations: AOD, alcohol and other drugs; AUDIT-C, 3-item Alcohol Use Disorder Identification Test; CUDIT-SF, Cannabis Use Disorder Identification Test–Short Form; HWC, health and wellness cultural workshop; TACUNA, Traditions and Connections for Urban Native Americans.

^a^ Missing for participants who reported no use in past 3 months at both baseline and 3-month follow-up.

^b^ Measured as 0 (never), 1 (hardly ever), 2 (sometimes), or 3 (often).

^c^ Measured from 0 (none) to 10 (100%).

^d^ Indicates 9-item Patient Health Questionnaire score of 10 or greater.

^e^ Indicates 7-item Generalized Anxiety Disorder Scale score of 10 or greater.

^f^ Cultural connection measured using the Sense of Community Index 2. Scores range from 0 to 3, with higher scores indicating more cultural connection.

### Baseline Attrition

Baseline characteristics are given in Table 1. There was no evidence of differential attrition in the CONSORT diagram (Figure). Among 279 individuals randomized to TACUNA, 186 (66.7%) received at least 1 workshop, and about half attended multiple sessions (1 session: 58 [20.8%]; 2 sessions: 36 [12.9%]; 3 sessions: 92 [33.0%]). In addition, 97 individuals (34.8%) attended a WC program, and 90 (92.8%) of those also attended at least 1 workshop. Among the 262 individuals randomized to the HWC group, 165 (63.0%) received the workshop.

**Figure.**
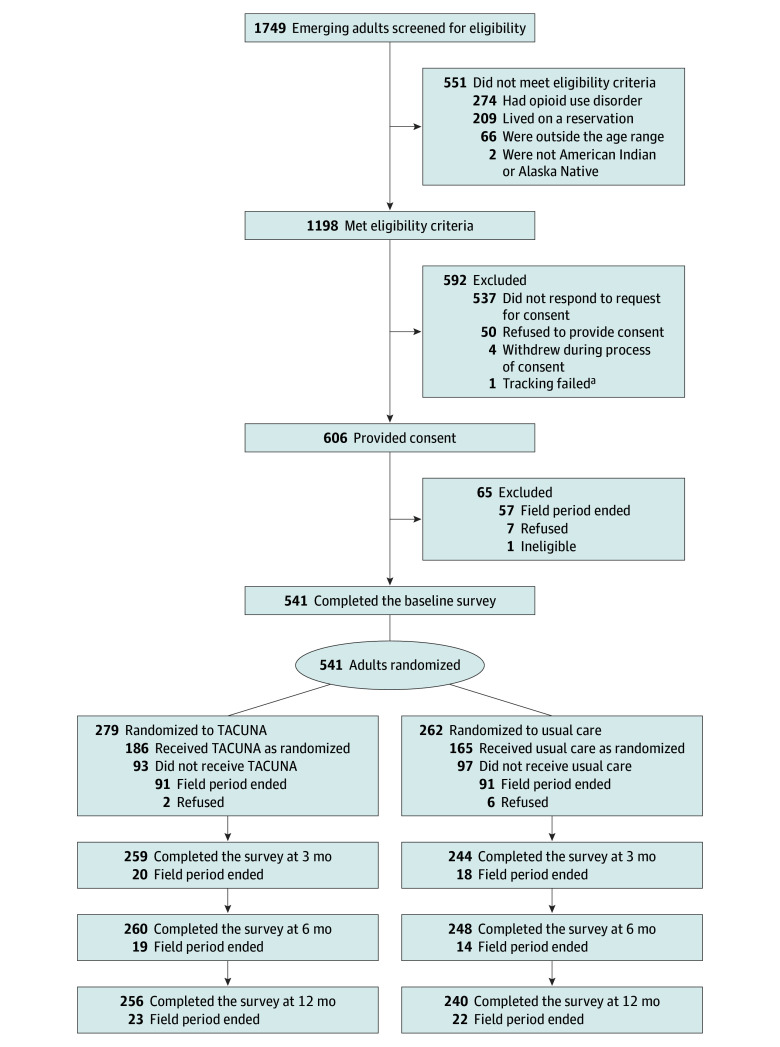
Study Flow Diagram TACUNA indicates Traditions and Connections for Urban Native Americans. ^a^Contact information was no longer viable and new contact information could not be obtained.

### Fidelity

Protocol fidelity was high in both conditions (eg, >90%). For TACUNA, all Motivational Interviewing Treatment Integrity global scale scores were above 4, indicating competency in MI.

### Alcohol

The largest number of alcoholic drinks consumed in 1 day significantly decreased for TACUNA (B [SE], −0.25 [0.09]; *P* = .003) but not HWC (B [SE], −0.15 [0.09]; *P* = .58) participants. Similarly, frequency of past 3-month alcohol use significantly decreased for TACUNA (B [SE], −0.51 [0.20]; *P* = .009) but not HWC (B [SE], −0.23 [0.18]; *P* = .21) participants. Negative alcohol-related consequences significantly and comparably decreased for both the TACUNA (B [SE], −0.99 [0.31]; *P* = .001) and HWC (B [SE], −1.01 [0.45]; *P* = .02) participants. Additionally, positive Alcohol Use Disorder Identification Test screens significantly decreased at similar rates for TACUNA (B [SE], −0.03 [0.01]; *P* = .005) and HWC (B [SE], −0.02 [0.01]; *P* = .02) participants (Table 2).

**Table 2.** Growth Parameters From Multigroup Latent Growth Models Across All 12-Month Outcomes

Outcome	HWC group				TACUNA intervention			
	Intercept		Slope		Intercept		Slope	
	B (SE)	*P* value	B (SE)	*P* value	B (SE)	*P* value	B (SE)	*P* value
Alcohol								
Largest No. of alcoholic beverages in 1 d (past 1 mo)	6.86 (0.26)	<.001	−0.15 (0.09)	.58	6.73 (0.23)	<.001	−0.25 (0.09)	.003
Use in past 3 mo	8.83 (0.62)	<.001	−0.23 (0.18)	.21	8.26 (0.57)	<.001	−0.51 (0.20)	.009
Negative consequences in past 3 mo	8.77 (1.37)	<.001	−1.01 (0.45)	.02	7.80 (1.08)	<.001	−0.99 (0.31)	.001
AUDIT-C positive screen	0.40 (0.03)	<.001	−0.02 (0.01)	.02	0.43 (0.03)	<.001	−0.03 (0.01)	.005
Cannabis								
Quantity per d^a^	1.10 (0.10)	<.001	0.01 (0.03)	.63	1.06 (0.09)	<.001	−0.07 (0.03)	.02
Cannabis use (past 3 mo)	10.62 (0.80)	<.001	−0.54 (0.21)	.01	9.36 (0.76)	<.001	−0.44 (0.20)	.03
Negative consequences (past 3 mo)	16.10 (1.96)	<.001	−2.22 (0.65)	.001	17.96 (2.12)	<.001	−1.74 (0.67)	.009
CUDIT-SF positive screen	0.32 (0.03)	<.001	−0.03 (0.01)	.005	0.29 (0.03)	<.001	−0.02 (0.01)	.02
Peer norms^b^								
Monthly alcohol use	7.19 (0.14)	<.001	−0.30 (0.06)	<.001	6.89 (0.14)	<.001	−0.30 (0.06)	<.001
Monthly cannabis use	6.54 (0.14)	<.001	−0.29 (0.06)	<.001	6.34 (0.14)	<.001	−0.36 (0.05)	<.001
Monthly prescription opioid use	2.71 (0.12)	<.001	−0.18 (0.04)	<.001	2.63 (0.12)	<.001	−0.22 (0.04)	<.001
Monthly heroin use	1.56 (0.09)	<.001	−0.09 (0.03)	.006	1.39 (0.08)	<.001	−0.07 (0.03)	.01
How often with peers who use AOD								
Alcohol	2.06 (0.05)	<.001	−0.04 (0.02)	.04	2.08 (0.05)	<.001	−0.08 (0.02)	<.001
Cannabis^a^	2.14 (0.06)	<.001	−0.04 (0.02)	.05	2.23 (0.06)	<.001	−0.10 (0.02)	<.001
Prescription opioids	0.43 (0.04)	<.001	−0.05 (0.02)	.001	0.44 (0.04)	<.001	−0.08 (0.01)	<.001
Heroin	0.13 (0.03)	<.001	−0.02 (0.01)	.07	0.16 (0.02)	<.001	−0.03 (0.01)	.001
Mental health								
Clinically meaningful anxiety^a^^,^^c^	0.37 (0.03)	<.001	−0.02 (0.01)	.03	0.38 (0.03)	<.001	−0.05 (0.01)	<.001
Clinically meaningful depression^d^	0.44 (0.03)	<.001	−0.04 (0.01)	<.001	0.39 (0.03)	<.001	−0.04 (0.01)	<.001
Cultural connectedness (sense of community)	1.68 (0.04)	<.001	0.02 (0.01)	.18	1.62 (0.04)	<.001	0.01 (0.01)	.22

Abbreviations: AOD, alcohol and other drugs; AUDIT-C, 3-item Alcohol Use Disorder Identification Test; CUDIT-SF, Cannabis Use Disorder Identification Test–Short Form; HWC, health and wellness cultural workshop; TACUNA, Traditions and Connections for Urban Native Americans.

^a^ Significant Wald test for slopes differences between groups.

^b^ Measured as the percentage of peers divided by 10.

^c^ Indicates 7-item Generalized Anxiety Disorder Scale score of 10 or greater.

^d^ Indicates 9-item Patient Health Questionnaire score of 10 or greater.

### Cannabis

Quantity of cannabis use per day significantly decreased among TACUNA (B [SE], −0.07 [0.03]; *P* = .02) but not HWC (B [SE], 0.01 [0.03]; *P* = .63) participants. Frequency of past 3-month cannabis use decreased in both TACUNA (B [SE],  −0.44 [0.20]; *P* = .03) and HWC (B [SE], −0.54 [0.21]; *P* = .01) participants. Similarly, cannabis-related negative consequences decreased in both TACUNA (B [SE], −1.74 [0.67]; *P* = .009) and HWC (B [SE], −2.22 [0.65]; *P* = .001) participants. Last, rates of positive Cannabis Use Disorder Identification Test–Short Form screens decreased in both TACUNA (B [SE], −0.02 [0.01], *P* = .02) and HWC (B [SE], −0.03 [0.01], *P* = .005) participants (Table 2).

### Peer Norms

Peer norms significantly improved in both groups. Specifically, perceived alcohol norms improved in both TACUNA (B [SE], −0.30 [0.06]; *P* < .001 and HWC (B [SE], −0.30 [0.06]; *P* < .001) participants; perceived cannabis norms improved in both TACUNA (B [SE], −0.36 [0.05]; *P* < .001) and HWC (B [SE], −0.29 [0.06]; *P* < .001) participants; perceived norms for monthly prescription opioid use improved in both TACUNA (B [SE], −0.22 [0.04]; *P* < .001) and HWC (B [SE], −0.18 [0.04]; *P* < .001) participants; and perceived monthly heroin use norms improved in both TACUNA (B [SE], −0.07 [0.03]; *P* = .01) and HWC (B [SE], −0.09 [0.03]; *P* = .006) participants (Table 2).

### Time With Peers Who Use Substances

Time spent around peers who use cannabis (B [SE], −0.10 [0.02]; *P* < .001) and heroin (B [SE],  −0.03 [0.01]; *P* < .001) significantly decreased during the 12-month period for TACUNA participants but not HWC participants (B [SE] for cannabis, −0.04 [0.02; *P* = .05]; B [SE] for heroin, −0.02 [0.01; *P* = .07], respectively). Time spent around peers who use alcohol decreased in both TACUNA (B [SE], −0.08 [0.02]; *P* < .001) and HWC (B [SE], −0.04 [0.02]; *P* = .04) participants. Similar outcomes were found for time spent around peers who used prescription opioids for TACUNA (B [SE], −0.08 [0.01]; *P* < .001) and HWC (B [SE], −0.05 [0.02]; *P* = .001) participants (Table 2).

### Mental Health

During the 12-month period, the positive screen rate for anxiety in the TACUNA group significantly decreased (B [SE], −0.05 [0.01]; *P* < .001), as in the HWC group (B [SE], −0.02 [0.01]; *P* = .03); however, the rate of decrease was significantly greater in the TACUNA group (Wald χ^2^ = 4.56; *P* = .03). Positive depression screen rates significantly and comparably decreased in both the TACUNA and HWC groups (B [SE], −0.04 [0.01]; *P* < .001 for both) (Table 2).

### Cultural Connection and Time for TACUNA Intervention

Cultural connectedness remained moderate and stable across the 12-month period in both TACUNA (B [SE], 0.01 [0.01]; *P* = .22) and HWC (B [SE], 0.02 [0.01]; *P* = .18) participants. Across all outcomes, there were no significant differences between the 1- and 2-hour versions of TACUNA for those who attended at least 1 workshop.

## Discussion

The present study is, to our knowledge, the first-large scale RCT testing 2 culturally grounded virtual interventions for substance use among urban American Indian and Alaska Native emerging adults across the US. More than 500 individuals from 37 states participated, highlighting that virtual recruitment and intervention were successful ways to engage this underserved and underresearched population.

Overall, both interventions decreased consequences of alcohol and cannabis use, depression, perceived norms, and time spent around peers using alcohol and prescription opioids. In addition, TACUNA participants reported greater decreases in quantity of cannabis use, time spent around peers using cannabis, and clinical anxiety compared with HWC participants, which is critical, as rates of AOD use and mental health problems are much higher among American Indian and Alaska Native emerging adults than other racial and ethnic groups.^8,10^ We found stable rates of cultural connectedness during the 12 months of follow-up in both groups, suggesting that these American Indian and Alaska Native emerging adults were able to maintain their connection with their communities.

Of note, the TACUNA 1-hour workshop version was as effective as the 2-hour version, increasing feasibility and sustainability. Overall, this type of virtually implemented culture-based program could benefit urban American Indian and Alaska Native communities in reducing alcohol and cannabis use disorders.

In sum, TACUNA was a brief, virtual, culturally grounded MI intervention specifically designed for this population that focused on participating in traditional practices, making healthy choices, and increasing supportive social networks, which may have led to greater decreases in AOD use and anxiety.^15,16^ Results are important for public health, as there are few cost-effective prevention resources available for urban American Indian and Alaska Native emerging adults that address substance use and mental health and also provide an opportunity to engage in culture with other American Indian and Alaska Native young people (unpublished data; S.M.,E.J.D., D.L.D., et al; 2026). Given the substantial barriers that many urban American Indian and Alaska Native people experience in accessing both prevention and cultural programming,^20,48^ TACUNA offered an innovative way to provide these services for this population.

### Limitations

This study has some limitations. First, it was limited in its generalizability, given the small number of male participants; however, prevention research with this age group and population typically has higher participation from females.^49^ Additionally, and not surprisingly, opioid use was extremely low, given our focus on prevention; thus, we were unable to statistically evaluate effects on opioid use. Furthermore, all data were self-reported; however, use rates in the sample were similar to national norms for alcohol use and higher for cannabis use.^47^ We provided cultural workshops to all participants, and in each condition 66.7% of participants received at least 1 workshop; however, the control condition only had 1 workshop whereas TACUNA had 3 workshops. Despite these limitations, this large RCT was the first, to our knowledge, to test 2 virtual cultural interventions among urban American Indian and Alaska Native emerging adults across the US, and our findings highlight how culturally grounded programming for this population can be protective for both substance use and mental health.

## Conclusions

In this RCT of urban American Indian and Alaska Native emerging adults, we found that participants in both groups reported reductions in consequences, norms, and time spent around peers using substances. Those who received TACUNA reported even lower quantity of cannabis use and clinical anxiety compared with those receiving the HWC condition, highlighting the important role of bringing urban American Indian and Alaska Native emerging adults together to discuss ways to reduce AOD use and socially connect with their tribal communities in the urban environment. Overall, our findings emphasize the critical role of virtual recruitment and service provision for this population as the study reached a large, diverse sample of urban American Indian and Alaska Native emerging adults across the US.

## Notes

Supplement 1.
Trial Protocol

Supplement 2. **eAppendix.** Fidelity Checklist Workshops

Supplement 3.
Data Sharing Statement
