# Supplementary material for: Virtual Culturally Grounded Interventions for Substance Use in Urban American Indian and Alaska Native Emerging Adults: A Randomized Clinical Trial
Source: JAMA Netw Open. 2026 Aug 11;9(8):e2627878. doi: 10.1001/jamanetworkopen.2026.27878 (PMC13463164; doi:10.1001/jamanetworkopen.2026.27878)
Supplement: Supplementary file 1 [file jamanetwopen-e2627878-s001.pdf]

Study Protocol and Statistical Analysis Plan

National Clinical Trials #: NCT04617938

Study Title: Traditions and Connections for Urban Native Americans (TACUNA)

Abbreviations

AI/AN	American Indian/Alaska Native
AOD	Alcohol and other drug
EA	Emerging adults
EAB	Elder Advisory Board
FG	Focus group
HEAL	Helping End Addiction Long Term
MICUNAY	Motivational Interviewing and Culture for Urban Native American Youth
OUD	Opioid use disorders
TACUNA	Traditions and Connections for Urban Native Americans
WC	Wellness Circle

Study Protocol

A clinical study or research protocol guides the study and associated data collection and analysis in a productive and standardized manner and is carefully designed to safeguard the participants' health and answer specific research questions. The protocol should describe the following aspects of the study.

1. What the study will do

This study responds to Request for Application-DA-19-035, HEAL (Helping End Addiction Long Term) initiative: Preventing OUD in Older Adolescents and Young Adults (ages 16-30) by developing and implementing a culturally centered intervention to address opioid use among urban American Indian/Alaska Native (AI/AN) emerging adults. The primary goal of this behavioral study is to compare AI/AN emerging adults who receive TACUNA (Traditions and Connections for Urban Native Americans) plus a Wellness Circle (WC) to those AI/AN emerging adults who receive an opioid education workshop on outcomes (e.g., opioid misuse and alcohol and other drug use) over a period of 12 months.

- TACUNA will be a virtual motivational interviewing group intervention that incorporates traditional practices and discussion of how to cultivate healthy social networks and cultural worlds. The Wellness Circle will be for emerging adults and people in their social network and will focus on how social networks and cultural connectedness influence healthy behaviors.
- Opioid education (control) will focus on discussion of opioid misuse within the AI/AN urban community and ways to reduce use in a culturally appropriate manner.

2. How it will be done

Phase 1/UG3

Aim 1. We will conduct focus groups (FGs) with emerging adults (EAs), parents of EAs, AI/AN providers, and our Elder Advisory Board (EAB) in urban communities throughout California to understand how to a) best identify, reach and engage AI/AN EAs to access programming addressing opioid use, b) adapt and enhance our existing culturally sensitive prevention intervention program, Motivational Interviewing and Culture for Urban Native American Youth (MICUNAY), for AI/AN EAs to address social network factors that amplify (or reduce) opioid and AOD use risk, and c) conduct a pilot test to ensure feasibility and acceptability of the TACUNA program.

Phase 2/UH3

Note: Due to the COVID-19 pandemic and restrictions on social gatherings, this study pivoted from in-person group implementation to virtual group implementation. This allowed us to expand participation beyond our original urban sites in California as we were no longer limited by facilitating in-person groups.

In Native American research, it is typical that intervention and control groups receive some degree of an intervention. We will enroll 370 participants at baseline across the United States who we determine represent the broader population of 18-25 year old urban AI/AN emerging adults. In a randomized controlled trial—an unmasked, 2-arm parallel intervention model—participants either will be assigned to TACUNA (n = 185), which comprises three virtual workshops utilizing motivational interviewing, social network visualization, and integrating traditional practices and a Wellness Circle (WC), or to one virtual culturally sensitive opioid education workshop (n = 185). WCs will be culturally focused, and feature Native foods and entertainment (e.g., Native lectures, storytellers, cooking demonstrations, and sage ceremony).

Aim 2. We will compare AI/AN EAs who receive TACUNA+WC to AI/AN EAs who receive opioid education. We will compare outcomes at 3, 6, and 12-months to determine (a) whether changes occur in initiation and escalation of opioid use and alcohol and other drug use, such as cannabis, and related consequences; time spent around peers who use opioids and AOD, and perceived prevalence of peer use, (b) whether clinically significant changes occur in physical, social, emotional, and functional well-being, as well as spirituality and cultural connectedness, and (c) if reductions occur, estimate effect sizes.

Aim 3. We will explore potential mechanisms of change for decreases in opioid and AOD use outcomes through mediation analyses, including changes in social networks and cultural connectedness.

Aim 4. We will develop and test strategies to facilitate sustainability of TACUNA within these communities through key informant interviews upon conclusion of the randomized controlled trial.

Aim 5. We will conduct an economic evaluation to quantify programmatic costs and cost-effectiveness of the multi-tiered intervention approach, relative to opioid education.

3. Why it is being done

This study's primary purpose is prevention. To date, there are no evidence-based prevention programs addressing opioid use among urban AI/AN emerging adults that integrate culturally appropriate strategies with evidence-based treatment. TACUNA builds on our prior work with AI/AN communities to develop and evaluate culturally appropriate programming to address opioid, alcohol, and cannabis use among urban AI/AN emerging adults.

Data from 2015 show that AI/AN people have the highest rates of diagnosis for opioid use disorders (OUD) and deaths from drug overdose. Unique risk factors may predispose urban AI/AN young adults to use opioids, alcohol or other drugs. For example, experiences of acculturative stress directly and indirectly associated with historical trauma experienced by AI/AN people throughout U.S. history result in poor health outcomes.

Programming that incorporates traditional practices, promotes community involvement, and encourages healthy notions of AI/AN identity may increase well-being and healthy behaviors by addressing sources of stress linked to cultural identity, stigma, and community connections. However, few evidence-based programs that integrate these cultural elements have been developed, implemented, and evaluated with urban AI/AN people using a strong research design. The TACUNA study substantially extends work with AI/AN emerging adults by adapting and testing an integrated culturally appropriate MI and social network intervention to address opioid and other AOD misuse at both the individual and community level.

4. How many people will be in the study

We will enroll 370 participants at baseline who we determine represent the broader population of urban AI/AN emerging adults. This sample size was determined in a priori power analyses to be sufficient to detect small to moderate intervention effect sizes for all primary and secondary outcomes.

5. Who is eligible to take part in it

Eligibility criteria include: 1) ages 18 to 25; 2) living in an urban area in any state in the United States (not on a rancheria or a reservation); 3) self-identification as AI/AN; 4) no opioid use disorder; and 5) English speaking.

6. What study therapy or other interventions will be given

None.

7. What tests will be done and how often

No tests will be done. Survey data will be collected at baseline, 3 months, 6 months and 12 months.

8. What information will be collected

From baseline to 3 months, to 6 months, and to 12 months, we will measure change in:

1. **Frequency of opioid use**. We will assess frequency of opioid use in the past three months from 1 = never to 6 = over 20 times.

2. **Frequency of alcohol and cannabis use**. We will assess frequency of opioid use in the past three months from 1 = never to 6 = over 20 times.

3. **Social Network Composition**. Participants will be asked to name 15 contacts ("alters") who are at least 18 years of age. Participants will rate each of the 15 people on the same set of questions with categorical response options (about demographics, relationship quality, likelihood to use drugs, etc.). For each participants the selected responses will be summed across all the network alters and divided by 15 to produce network composition percentages of the whole set of alters named by the participant (% of AI/ANs, % who engage in heavy drinking).

4. **Cultural connection**. Cultural connection will be measured with 29 items that address 3 dimensions: identity, traditions, and spirituality. Respondents answer 11 yes/no questions (e.g., I have a traditional person, Elder, or other person who I talk to), and use a scale from 1= "strongly disagree" to 5= "strongly agree" for 18 items (e.g., I feel a strong connection/attachment towards my Native American community or Tribe). We will also measure this via participation in 26 different types of traditional practices (e.g., going to Pow Wows, beading, traditional dancing). Items were based upon extensive research conducted with AIAN young people, parents, and community partners.

5. **Social Network Structure**. Participants will rate the relationship strength between each of the 15 named alters. For each alter pair they will rate if the two people know each other and, if yes, have they connected recently. Measures of network structure (i.e. network "connectedness") will be constructed from the set of evaluations for each participant. For example, network "density" will be constructed for the network overall, which is calculated by summing the number of alter pairs who know each other and dividing by the total number of possible ties among 15 alters (105). Individual alter "centrality" will be calculated by summing the number of connections each individual alter has with other alters in the network.

Statistical Analysis Plan (SAP)

The statistical analysis plan outlines a proposed method to describe and define the following aspects of the study.

1. Statistical aspects of the clinical trial design

Aim 2. We will compare outcomes at 3-, 6- and 12-months for AI/AN EAs who receive opioid education (n=185) to AI/AN EAs who receive TACUNA+WC (n=185).

Aim 3. We will conduct mediation analyses to examine whether changes in social networks and cultural connectedness serve as explanatory mechanisms for reductions in opioid and AOD use.

Aim 4. Sustainability. We will develop and test strategies to facilitate sustainability of TACUNA within these communities through 20 key informant interviews.

Aim 5. Economic evaluation. We will follow recommendations from the Panel of Cost-Effectiveness in Health and Medicine, by reporting costs and cost-effectiveness estimates in terms of two reference cases, one based on a program perspective (programmatic and provider time costs) and the other on a societal perspective that incorporates a broader range of costs, such as opportunity costs to participants.

2. Process of data selection for analyses

Data were selected for analysis in order to address study aims focused on evaluating the intervention in terms of primary and secondary outcomes as well as determining mediating mechanism of change.

3. Detailed analyses of data items

Descriptives. We will examine descriptive statistics and examine missing data. Frequencies will be evaluated for evidence of sparseness for categorical data and for non-normality (using plots, examination of skewness, kurtosis, etc.) for continuous variables. If sparseness is present for categorical variables, we will collapse as necessary to produce cell sizes sufficient for analysis. Where non-normality is evident, variables may be transformed. Outliers may be recoded or omitted if necessary. Missing data will be dealt with using multiple imputation and/or full information maximum likelihood estimation. All outcomes will be presented using descriptive statistics; continuous variables by the mean and standard deviation (SD), and dichotomous variables by the number and percentage. Descriptive statistics will be provided at both baseline and follow-up.

Baseline equivalence across experimental groups. We will evaluate comparability of experimental groups with respect to potential confounders. Categorical methods of analysis (e.g. cross tabulations, chi-square) will be used to compare groups for discrete data (e.g., employment, school status). ANOVA or t-tests will be used to test for homogeneity of groups for continuous data at baseline. If a statistically significant difference is found, the covariates will be included in all subsequent analyses.

4. Procedures and methods employed for analyzing the data

Aim 2

Analysis. Our analytic approach will focus on use and consequences, cognitions, social networks, and cultural connection outcomes. We will compare opioid education participants to TACUNA+WC participants, with covariates age, gender, and the baseline measure of the outcome of interest. Given the longitudinal design, we may implement more than one analytic method to address this aim. One option is to use a multigroup latent growth model framework using robust maximum likelihood for continuous outcomes or weighted least square mean and variance for categorical outcomes. Alternatively, we may use SAS Proc Mixed will be used for continuous outcomes and SAS Proc Glimmix/NLMixed for categorical outcomes. In addition to examining overall longitudinal trends across the entire study, we will also examine outcomes are each timepoint (3, 6, and 12 month) using multivariable linear regression for normally distributed outcomes and multivariable logistic regression for dichotomous outcomes, controlling for the baseline value of the outcome and participant demographics (age, sex, and sexual and gender diverse identity). The estimated adjusted difference between intervention groups at follow-up (or odds ratio for dichotomous outcomes) and the corresponding 95% confidence interval (CI) will be presented, along with the p-value and Cohen's effect size. Evaluating differences at each timepoint provides estimates of the intervention effect at a given timepoint whereas the longitudinal methods will address patterns of change over the course of the intervention. To enhance interpretability of our results, we will use estimated differences between groups to calculate standardized effect sizes in terms of *d* (for continuous variables, using the standard deviation of the opioid education only group as denominator); we will use odds ratio estimates to calculate risk difference and number needed to treat, based on outcome proportions for the opioid education group.

Power. The N of 370 was determined in a priori power analyses to be sufficient to detect small to moderate intervention effect sizes for all primary and secondary outcomes. We chose to use the final projected sample size accounting for 20% attrition at the 12-month at follow up (n=148 per arm). Within a repeated measures framework, these sample sizes, assuming a correlation between repeated assessments of 0.50, four timepoints, and alpha of .05, we have 80% power to detect a standardized effect size (d) of 0.31 between groups and .22 within groups. For mediation models, using bias-corrected bootstrapping, there is 80% power to detect a mediated/indirect effect when $\delta=0.28$ (i.e., effect of the predictor on the mediator) and $\beta=0.26$ (i.e., effect of the mediator on the outcome).

Aim 3

Mediation models will be used to determine whether changes in cultural connectedness and social networks serve as mechanisms for reducing opioid, cannabis, and alcohol use. First we will test multivariate mediation to determine if the total mediation effect is statistically significant to control Type I error rate. If the total mediated effect is significant, we will then examine individual mediators to determine statistical significance. The estimate of the effect of the intervention on the outcome can be divided into the direct effect and the indirect (mediated) effect. The standard errors of the individual indirect effects will be estimated using bias-corrected bootstrapping. For mediation models, using bias-corrected bootstrapping, with these projected sample sizes, there is 80% power to detect a mediated/indirect effect when $\delta=0.28$ (i.e., effect of the predictor on the mediator) and $\beta=0.26$ (i.e., effect of the mediator on the outcome). Results will provide information about efficacy for each intervention. Results can improve the design of future studies by providing information such as intra-class correlations within groups, which will allow researchers to estimate sample sizes and effect sizes for power analysis.

Aim 5

Costs. We will account for programmatic costs following the micro-costing approach of prior work.

Intervention costs will be obtained from several sources, including program records and primary collection of cost and time use data. Programmatic input costs include labor (including overhead), materials, and other costs of implementing the intervention; for the societal perspective, we will also consider (a) transportation costs to participants; (b) opportunity costs of EAs; and (c) space and utility costs. The analysis will exclude research costs, although we can incorporate these in sensitivity analyses.

Effectiveness. We will follow recommendations for economic evaluations of AOD use interventions and consider multiple outcomes from Aim 2 analyses to compare effectiveness at 6 and 12 months after baseline. Our primary effectiveness measures will be abstention from opioid misuse in the past 3 months (either through preventing initiation or discontinuing use); abstention or consumption below the threshold of heavy use for alcohol and cannabis; and a reduction in time spent around peers who misuse opioids. Given our focus on prevention, an effectiveness outcome related to peers will be informative.

Economic analysis. We will conduct an intent-to-treat analysis, aligned with the primary analytic plan of Aim 2. We will first perform deterministic analyses (i.e., direct calculations) to sum program costs (and opportunity costs for the societal perspective), yielding incremental net costs as the difference between net costs per participant to (a) those receiving TACUNA+WC and (b) those receiving opioid education. Total costs and costs per participant will be calculated separately by site to assess whether key factors determine cost variability for each intervention. Next, we will perform probabilistic (Monte Carlo) analyses to account for uncertainty and variability in cost parameters. Incorporating Aim 2 findings for each of our primary effectiveness outcomes, we will construct short term incremental cost-effectiveness ratios (ICERs) for each outcome. We will assess model and parameter uncertainty on each outcome's effect sizes, allowing us to consider a range of values from findings related to uncertainty or variability in the relative effectiveness of TACUNA+WC compared to opioid education. We will construct ICER confidence intervals using nonparametric bootstrapping techniques within the multivariable framework, although we may consider other methods.

Sensitivity analysis. We will perform sensitivity analyses to test the importance of key model parameters, such as the price of a given resource, on ICER findings. Additionally, we will consider how costs and cost-effectiveness differ under alternative parameter assumptions as well as under different assumptions about attendance.

5. Planned presentation of results in formats such as tables, listings, and figures

Results will be depicted in tables or figures as appropriate.

6. Plans for interim and final analysis and statistical analysis of the primary and secondary variables and other data

All analyses detailed above in Section 3 including timepoint specific follow-up as well as overall longitudinal models will compare intervention conditions on change in primary outcomes. Mediation models will include secondary variables (social networks and cultural connectedness) as potential explanatory mechanisms of change.
