# Supplementary material for: Virtual Culturally Grounded Interventions for Substance Use in Urban American Indian and Alaska Native Emerging Adults: A Randomized Clinical Trial
Source: JAMA Netw Open. 2026 Aug 11;9(8):e2627878. doi: 10.1001/jamanetworkopen.2026.27878 (PMC13463164; doi:10.1001/jamanetworkopen.2026.27878)
Supplement: Supplementary file 2 [file jamanetwopen-e2627878-s002.pdf]

# Supplementary Online Content

D'Amico EJ, Dickerson DL, Rodriguez A, et al. Virtual culturally grounded interventions for substance use in urban American Indian and Alaska Native emerging adults: a randomized clinical trial. *JAMA Netw Open.* 2026;9(8):e262787. doi:10.1001/jamanetworkopen.2026.27878

**eAppendix.** Fidelity Checklist Workshops

This supplementary material has been provided by the authors to give readers additional information about their work.

© 2026 D'Amico EJ et al. *JAMA Network Open*.

**eAppendix.** Fidelity Checklist Workshops

**Fidelity Checklist Workshop 1 (Behavioral Dimension)**

Below are elements of the TACUNA Workshop 1 (e.g., points of information, questions to elicit discussion, group activities) which the facilitator is expected to deliver for Workshop 1. The facilitator is not required to say verbatim the statements and questions below; however, s/he must adequately convey the same message. **Put an X next to each element that you observe the facilitator delivering during this session.**

***note: in this workshop, the opening prayer will be done at the beginning even though the cultural activity is in Part II**

**Part I**

- ∆ Starts with **OPENING PRAYER**
- ∆ Discusses counselor **PHILOSOPHY** (e.g., safe place to talk about different issues; if there is any changing to be done, that is up to you; not here to tell you what to do)
- ∆ Reviews **CONFIDENTIALITY** (e.g., what is discussed is confidential unless we need to protect you or others from harm)
- ∆ Generates **RULES** with the group
- ∆ Discusses **SOCIAL NETWORKS AND CHOICES**
    - *For example, walks participants through the social network diagram components; prompts participants to refer to their own social network diagram; fosters conversation about social networks and finding support*
- ∆ Discusses **SOCIAL RELATIONSHIPS AND THE BRAIN**
- ∆ Conducts **MEMORY GAME WITHOUT AND WITH DISRUPTION**
- ∆ Discusses how **DIFFERENT SUBSTANCES (E.G., OPIOIDS, ALCOHOL, MARIJUANA) AFFECT** the brain
- ∆ Discusses continued use of alcohol, opioids, and other drugs on the brain including **TOLERANCE**
- ∆ Discusses how the brain **RECOVERS**
- ∆ Asks **HOW INFORMATION AFFECTS THEIR CHOICES**

**Part II**

- ∆ Discusses **MEDICINE WHEEL**
- ∆ Discusses **HISTORICAL TRAUMA AND CULTURAL IDENTITY**
- ∆ Discusses **GATHERING OF NATIONS** principles
- ∆ Discusses how doing **ENJOYABLE TRADITIONAL ACTIVITIES AFFECTS** the brain
- ∆ Discusses **STORYTELLING** and
    - Shows **AT LEAST ONE STORYTELLING VIDEO**
- ∆ Asks **HOW THEY MIGHT TELL THEIR OWN STORY**
- ∆ Ends with **SEND-OFF PRAYER**

© 2026 D’Amico EJ et al. *JAMA Network Open*.

**Fidelity Checklist Workshop 2 (Physical Dimension)**

Below are elements of the TACUNA Workshop 2 (e.g., points of information, questions to elicit discussion, group activities) which the facilitator is expected to deliver for Workshop 2. The facilitator is not required to say verbatim the statements and questions below; however, s/he must adequately convey the same message. **Put an X next to each element that you observe the facilitator delivering during this session.**

***note: in this workshop, the opening prayer will be done at the beginning even though the cultural activity is in Part II**

**Part I**

- Δ Starts with **OPENING PRAYER**
- Δ Discusses counselor **PHILOSOPHY** (e.g., safe place to talk about different issues; if there is any changing to be done, that is up to you; not here to tell you what to do)
- Δ Reviews **CONFIDENTIALITY** (e.g., what is discussed is confidential unless we need to protect you or others from harm)
- Δ Generates **RULES** with the group
- Δ Discusses **PROS AND CONS OF AOD USE**
- Δ Discusses **SOCIAL NETWORKS AND CHOICES**
- Δ Discusses **HOW TO DECREASE CONSEQUENCES AND GET POSITIVES WITHOUT USING**
- Δ Goes over **PATH OF CHOICES HANDOUT**
- Δ Discusses **EXPERIMENTAL, PROBLEM USE, AND ADDICTION**
- Δ Discusses **WILLINGNESS AND CONFIDENCE RULERS**

**Part II**

- Δ Discusses **MEDICINE WHEEL**
- Δ Discusses **TRADITIONAL FOODS**
- Δ **Discusses FOOD AS OUR MEDICINE**
- Δ Goes over **TRADITIONAL FOOD HANDOUTS**
- Δ Shows **SUGGESTED NATIVE COOKING VIDEOS**
- Δ Discusses **VIDEO CONTENT**
- Δ Ends with **SEND-OFF PRAYER**

© 2026 D'Amico EJ et al. *JAMA Network Open*.

**Fidelity Checklist Workshop 3 (Spiritual Dimension)**
Below are elements of the TACUNA Workshop 3 (e.g., points of information, questions to elicit discussion, group activities) which the facilitator is expected to deliver for Workshop 3. The facilitator is not required to say verbatim the statements and questions below; however, s/he must adequately convey the same message. **Put an X next to each element that you observe the facilitator delivering during this session.**

***note: in this workshop, the opening prayer will be done at the beginning even though the cultural activity is in Part II**

**Part I**

- Δ Starts with **OPENING PRAYER**
- Δ Discusses counselor **PHILOSOPHY** (e.g., safe place to talk about different issues; if there is any changing to be done, that is up to you; not here to tell you what to do)
- Δ Reviews **CONFIDENTIALITY** (e.g., what is discussed is confidential unless we need to protect you or others from harm)
- Δ Generates **RULES** with the group
- Δ Discusses **WHAT CAN HAPPEN WHEN PEOPLE USE AOD**
- Δ Discusses **SOCIAL NETWORKS AND CHOICES**
    - *For example, prompts Workshop 1 participants to describe the social network diagram and resulting discussion; fosters conversation about social networks and finding support*
- Δ Discusses **UNPROTECTED SEX, STD, AND HOW TO AVOID**
- Δ Goes over **HIV QUIZ**
- Δ Does **WHEEL OF THE FUTURE GAME**
- Δ Discusses **WILLINGNESS AND CONFIDENCE RULERS**

**Part II**

- Δ Discusses **MEDICINE WHEEL**
- Δ Discusses **RESPONSIBLE SAGE HARVESTING**
- Δ Does **SAGE AND SMUDGING TRADITIONAL ACTIVITY**
- Δ **Discusses THE ROLE OF SPIRITUALITY IN ONE'S LIFE**
- Δ Shows **SUGGESTED SMUDGING VIDEOS**
- Δ Discusses **VIDEO CONTENT**
- Δ Ends with **SEND-OFF PRAYER**

© 2026 D'Amico EJ et al. *JAMA Network Open*.
