# Supplementary material for: Virtual Culturally Grounded Interventions for Substance Use in Urban American Indian and Alaska Native Emerging Adults: A Randomized Clinical Trial
Source: JAMA Netw Open. 2026 Aug 11;9(8):e2627878. doi: 10.1001/jamanetworkopen.2026.27878 (PMC13463164; doi:10.1001/jamanetworkopen.2026.27878)
Supplement: Supplementary file 3 [file jamanetwopen-e2627878-s003.pdf]

Data Sharing Statement

D'Amico. Virtual Culturally Grounded Interventions for Substance Use in Urban American Indian and Alaska Native Emerging Adults. *JAMA Netw Open*. Published August 11, 2026. doi:10.1001/jamanetworkopen.2026.27878

Data
Additional Information: ClinicalTrials.gov Identifier: NCT04617938. Registered October 26, 2020 https://clinicaltrials.gov/ct2/show/record/NCT04617938
Data available: Yes
Data types: Deidentified participant data
How to access data: Upon study completion, data will be available through the National Addiction & HIV Data Archive Program (NAHDAP).
When available: With publication

Supporting Documents
Document types: None

Additional Information
Who can access the data: researchers whose proposed use of the data has been approved.
Types of analyses: for a specified purpose
Mechanisms of data availability: with a signed data access agreement
Any additional restrictions: after approval by our Urban Intertribal Native American Review Board
